# Innovative Hemp Shive-Based Bio-Composites, Part II: The Effect of the Phase Change Material (PCM) Additive on Characteristics of Modified Potato Starch Binders

**DOI:** 10.3390/ma18040891

**Published:** 2025-02-18

**Authors:** Laura Vitola, Ina Pundiene, Jolanta Pranckevičienė, Diana Bajare

**Affiliations:** 1 Institute of Sustainable Building Materials and Engineering Systems, Riga Technical University, Kipsalas 6A, LV–1048 Riga, Latvia; 2 Laboratory of Concrete Technologies, Institute of Building Materials, Vilnius Gediminas Technical University, Sauletekio al. 11, LT–10223 Vilnius, Lithuania

**Keywords:** potato starch binder, hemp shives, bio-composite, sustainable building materials, phase change materials, bio-composites

## Abstract

This study investigates the effect of phase change materials (PCM) on the properties of modified potato starch binders and hemp shive-based bio-composites, emphasizing their potential for sustainable construction applications. PCM-modified binders have shown reduced viscosity during gelatinization, enhancing their workability and uniformity during processing. A microstructural analysis reveals that PCM addition results in a denser and more cohesive binder network, leading to improved adhesion and reduced porosity. A thermal analysis demonstrates a shift to higher decomposition temperatures and a linear increase in specific heat capacity within the PCM phase-change range (20–30 °C), significantly enhancing the thermal storage capacity of the bio-composites. PCM addition improves compressive strength by up to twice, with optimal performance achieved at 8% PCM additive content. The prolonged cooling time, up to three times longer in bio-composites with PCM additive, highlights their effectiveness in thermal regulation. Additionally, bio-composites with a PCM additive exhibits increased bulk density and reduced water swelling, improving dimensional stability. These findings underline the dual benefits of enhanced thermal and mechanical performance in bio-composites with a PCM additive, making them a viable alternative to conventional building materials.

## 1. Introduction

Buildings’ heating and cooling are thought to account for one-third of building energy use. The energy needed for cooling will increase by 150% by 2050 [1,2]. The energy demand for building cooling in emerging countries is expected to increase by 300% to 600% by 2050. Enhancing indoor thermal comfort and reducing the energy required for space cooling are two benefits of optimizing thermal efficiency in building components [3]. The intended level of thermal comfort can be attained by enhancing these building envelopes’ thermal performance, increasing the walls’ inertia and thermal resistance [2]. However, the design constraints of wall thickness and weight suggest that it is very difficult to achieve the required levels of thermal resistance and inertia increase by traditional solutions. Recently developed phase change materials (PCMs) have a high specific latent heat capacity. For maximum effectiveness, a PCM, which is also used in energy efficient building solutions, should melt and solidify between 18 and 30 °C. Compared to traditional (non-PCM) materials, these materials have a 15-fold greater capacity to absorb and release thermal energy [4]. Adding PCMs to these building envelopes can also increase their thermal inertia and storage capacity, which will improve their thermal performance [5]. It has recently been confirmed that integrating a PCM into building envelopes can save energy consumption for heating and cooling while improving thermal comfort [6,7]. However, the incorporation of a PCM into the composite has a lot of challenges. A recent article highlights the technique of integrating a PCM into building structures. There are still serious design challenges of the used binders [7], compatibility with composite components, and another challenge—which is how to integrate the PCM into the building envelope along with where and how thick it should be placed in the building wall. A few attempts were made to include a PCM in bio-based construction materials [2,8]. PCM microencapsulation is one of the most common ways of introducing a PCM in newly constructed building envelopes without the deterioration of the base material. There were some efforts to incorporate a PCM in bio-based building materials.

The creation of reusable construction materials with a high waste content is a crucial area of research for waste disposal as a resource. The lack of green infrastructure in urban areas has been strongly linked to an increase in resident health issues, making the sustainable building envelope especially important [5]. Natural fillers can be derived from agricultural by-products such as hemp shives [9,10] and combined with natural source starch as a binder [11]. Recent studies have shown that starch is a great alternative to traditional, inorganic binders. Starch is a noteworthy resource for a variety of bio-composite applications due to its vast availability, low cost, and perfect composting ability without leaving any harmful residues [7,12,13]. When compared to bio-composites created using binders based on cement, lime, clay, or magnesium binder, the starch-based bio-composites had better thermal insulating qualities due to their low density and high porosity (around 89%) [13,14]. The most current breakthroughs in starch modification, applying sodium silicate and glycerol for employment as a key component in industrial bio-composite materials, are described in research [11].

Hemp-based materials reduce surface heat and improve thermal comfort. Therefore, by passively controlling the interior temperature, the use of such materials effectively reduces the building’s energy usage [15]. The thermal properties of a bio-composite can be improved with PCM addition [16]. The density of such a composite reaches 350 kg/m^3^, its mechanical strength maximally strives for 1.2 MPa, and its thermal conductivity values do not go above 0.075 W/(m·K) [17,18]. During the mixture production process, the samples’ specific heat capacity value increased by 62% when 5% (dry matter) of the PCM was added to the composition. Despite PCMs’ promise for thermal energy storage in buildings, there are a few possible drawbacks that should be considered. One issue with using PCMs is the cost. Not only are PCMs more costly than traditional insulating materials, but integrating them into building structures may also need additional engineering and construction labor. Another challenge is PCMs’ poor heat conductivity [19,20,21].

This study is a follow-up to a previous study that investigated the modification of a potato starch binder with sodium meta-silicate solution and glycerol and the use of these binders in the production of bio-composites [11]. In Part II, the investigation focuses on the effect of a PCM additive on the properties, including the rheological behavior of a fresh binder and mechanical compressive strength of bio-composites, of both a potato starch binder modified with sodium metasilicate and glycerol and hemp-based bio-composites.

## 2. Materials and Methods

### 2.1. Raw Materials

In this study the main raw materials for the binder were as follows:

- Potato starch (Ltd. Aloja Starkelsen, Ungurpils, Latvia) with the bulk density 595 kg/m^3^, amylose content 26.9%, moisture content 15.1%, and gelatinization temperature 64 °C;
- Sodium metasilicate solution (Na_2_SiO_3_ · xH_2_O,) (Ltd. Vincents Polyline, Kalngale, Latvia) with density of 1.37 g/cm^3^, molar SiO_2_/Na_2_O ratio of 3.22, and boiling point of 100 °C;
- Glycerol (C_3_H_8_O_3_) (Ltd. Farmacom, Kharkiv, Ukraine) with density of 1.260g/cm^3^; a refractive index of 1.47; and the melting point and boiling point, respectively, at 17.9 °C and 290 °C.

Additionally, in this study, the potato starch binder was modified by MikroCapsPCM25-S50—formaldehyde-free, concentrated aqueous dispersion of microencapsulated paraffin wax, also known as phase change material (PCM) (Ltd. MicroCaps, Ljubljana, Slovenia) [22].

To prepare the bio-composites for this study, the hemp shive aggregate (UPB Naturalus pluostas, Kedainiai, Lithuania) resulting from a hemp fiber separation process was used [23].

### 2.2. Compositions and Preparation of Samples

To be able to analyze more accurately the impact of the modification of potato starch binder by PCM on the properties of resulting bio-composites, first, the samples of studied binders were prepared in accordance with Table 1. All studied binder compositions consisted of same amount of potato starch, sodium metasilicate solution, glycerol, and water, while PCM amount was variable factor.

In Table 2, the compositions of the studied bio-composites are given. To characterize the PCM impact on the resulting material (respectively, bio-composite) properties, all raw materials, except the PCM additive, were present in constant quantities in the samples produced. The amount of PCM varied from 0% to 32% of the potato starch content.

**Figure 1:**
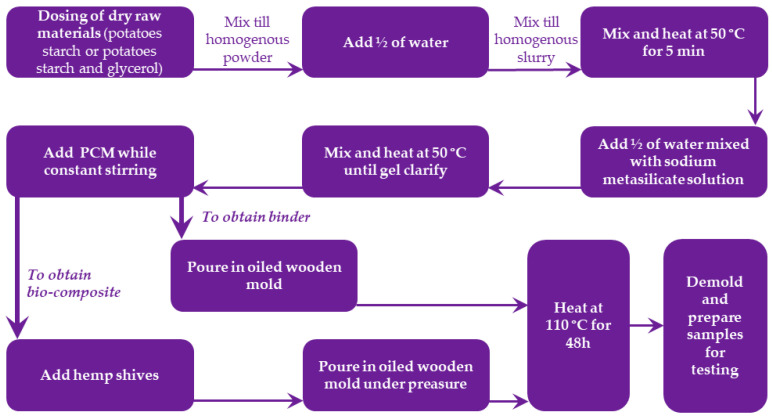
Preparation scheme of studied binders and bio-composites.

Both the binder and the bio-composite samples were prepared according to the scheme illustrated in Figure 1. The PCM additive was added to the composition in the last step of preparation of binder, and the rest of the preparation procedure followed was analogous to that used in the first part of the study described in Part I [11].

The preparation process began with the dosing of dry raw materials, such as potato starch or a mixture of potato starch and glycerol, followed by thorough mixing until a homogeneous powder was obtained. Half of the required water was then added to the mixture, and the components were stirred to form a uniform slurry. This slurry was heated to 50 °C while mixing continuously for 5 min. The remaining water, pre-mixed with sodium metasilicate solution, was gradually added, and the mixture was further heated at 50 °C until the gel clarified. At this stage, the PCM was added under constant stirring to obtain the binder; compositions of studied binders are given in Table 1. Once the binder was prepared, it was poured into an oiled wooden mold.

Meanwhile to produce bio-composites, hemp shives were incorporated into the binder mixture before pouring into the mold. In this case, the mixture was subjected to pressure while placed in the oiled wooden mold. The exact compositions of the bio-composites are detailed in Table 2.

The samples, both binder and bio-composites, were then cured by heating at 110 °C for 48 h. After the curing process, the samples were demolded and prepared for testing.

### 2.3. Testing Methods

The viscosity of fresh binders was tested by SV10 Vibro-viscometer (A&D COMPANY, Tokyo, Japan).

Studied binder samples were characterized by the thermogravimetric analysis (TGA) and differential scanning calorimetry (DSC) using METTLER TOLEDO TGA/DSC 3+, STARe System thermobalance. Samples were tested using 10 °C/min heating speed up to 600 °C in an atmosphere.

To determine the types of chemical bonds present in the studied binders, Fourier transform infrared spectroscopy (FTIR) spectra with a range of 4000–400 cm^−1^ were obtained with the Varian FTS 800 FT-IR Scimitar Series (Varian, Palo Alto, CA, USA) spectrometer using test samples containing 300 mg of KBr and 1 mg of the respective binder.

The microstructure of both binders and bio-composites were performed with the scanning electron microscopy (SEM) using Mira/LMU (Tescan, Brno, Czech Republic).

The bulk density of the studied bio-composites was determined in accordance with the standard EN 1602 [24] but with capillary water absorption—EN 1609 [25] (method A).

The mechanical strength in compression of studied bio-composites was determined at 10% and 20% of deformation accordance with the standard EN 826 method [26] using H10KS (Hounsfield, Surrey, UK) computerized machine (max load—10 kN, loading accuracy—±0.5%, loading speed accuracy—±0.05%).

The thermal conductivity of studied bio-composites was determined in accordance with the standard EN 12667 [27] using the LaserComp FOX 304 (measurement limits 0.01–0.50 W/(m·K), measuring accuracy ~1%). Measurements were obtained with 20 °C difference between measuring plates, with average test temperature—10 °C.

## 3. Results

### 3.1. The Behavior of Binders

Like the previous investigation described in Part I [11], properties of selected binders (Table 1) were determined to characterize the influence of the PCM additive on the material properties, excluding the influence of the aggregates (hemp shive).

**Figure 2:**
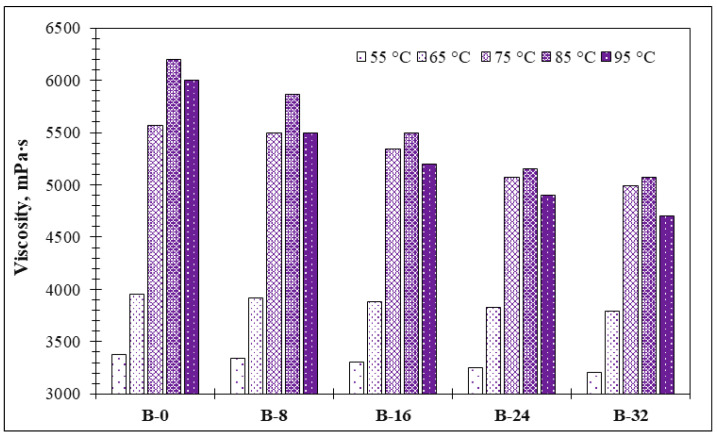
Viscosity of obtained potato starch binder.

Gelatinized starch viscosity is one of the major parameters that enables to estimate the behavior of slurry during heating and further mixing with aggregates. When heated in water, starch undergoes a transition process, during which the granules break down into a mixture of polymers-in-solution, known as gelatinization. Functional properties of starch are directly influenced by hydrothermal (heat and moisture) treatment or processing conditions. When raw starch granules are heated in water, the semi-crystalline nature of their structure is reduced or eliminated and the granules break down, forming a viscous solution; solution viscosity depends on starch source and concentration. Heat-induced starch granule breakdown in water is known as gelatinization [28]. Gelatinization starts with a pasting temperature and the hydration of starch granules, then the maximal intensity of gelatinization appears with the highest viscosity. After this destruction of starch granules, the complete dispersion with the start of the drop of viscosity is observed. The gelatinized slurry viscosity tests showed that the viscosity of the slurry depends on the amount of the PCM and heating temperature (Figure 2). In control composition B-0, after the slurry reaches 55 °C, the viscosity reaches 3376 mPa∙s, while it is lower in slurries with the PCM. A higher amount of the PCM leads to viscosity up to 6% lower than in the control sample. The gelatinization is initiated by the absorption of water into the granules leading to the hydration of the amorphous shell and the simultaneous disruption of hydrogen bonds. Continuing heating includes the melting of starch crystallites, starch solubilization, and the leaching out of starched granules, which can be seen in a loss of birefringence and increase in suspension viscosity [28,29]. When the heating temperature reaches 65 °C, the difference between viscosities in the control sample and samples with the PCM increases. In control composition B-0, the viscosity reaches 4500 mPa∙s, whereas in samples with the PCM, it is 4.5, 11.8, 13.3, and 13.9% lower. As is pointed out in several publications [30,31], the addition of a PCM or salts decreased viscosity. A further increase in temperature until 75 °C leads to additional growth in viscosity compared to the control sample, for which the viscosity reaches 5770 mPa∙s. In samples with the PCM, the viscosity is 2.9, 7.3, 12.0, and 13.5% lower than in the control sample. The temperature of 85 °C leads to the peak viscosity values of gelatinized starch. In the control sample, it reaches 6120 mPa∙s; in samples with the PCM, the viscosity values are lower—5870, 5510, 5152, and 5071 mPa∙s—and it means that viscosity is up to 17% lower than in the control sample. At 95 °C, the viscosity starts to drop, which illustrates that maximal gelatinization is finished. The B-32 sample shows a significant drop in viscosity, being 21.6% lower compared to the viscosity of the control sample (i.e., B-0). This result shows that the PCM lowers the viscosity of gelatinized starch and that it is a positive factor in using gelatinized starch when mixing with aggregates.

**Figure 3:**
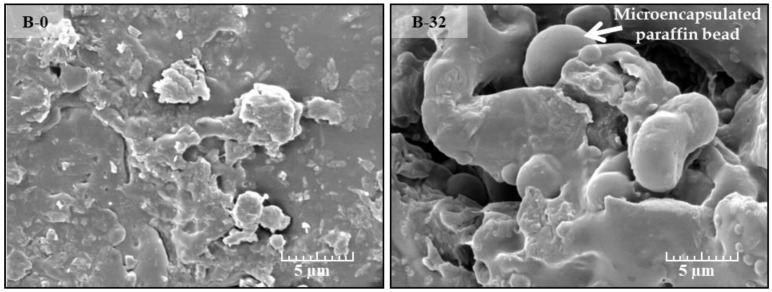
Micro-structure of B-0 and B-32.

The obtained SEM images (Figure 3) reveal microstructural differences between the two samples of a potato starch-based binder. Reference binders for sample B-0 (Figure 3, on the left) consist of potato starch modified with sodium metasilicate and glycerol. However, B-32 (Figure 3, on the right) is a potato starch binder modified with sodium metasilicate and glycerol with the addition of the phase change material (PCM).

The microstructure of the B-0 sample appears relatively loose and less compact compared to B-32. Many small, irregularly shaped particles are scattered throughout the matrix, and gaps and pores are also observed. The surface morphology suggests that there is no strong bonding between the particles, resulting in a more porous structure. This morphology is indicative of a lower binder density and/or cohesion in the matrix, which can directly affect mechanical properties, such as compressive strength and durability.

The effect of the PCM additive on the change in the structure of the binder is clearly visible. The structure of B-32 is denser in the form of a more interconnected network with larger, smooth, spherical formations, identifiable as PCM spheres. The PCM is partially encapsulated and integrated into the starch matrix. The structure of B-32 suggests that the addition of the PCM reduces the porosity of the binder, making the structure denser. In the case of potato starch-based bio-composites, the addition of the PCM may not only improve the thermal performance due to the ability of the PCM to absorb and release heat but may also improve the mechanical properties by providing a more homogeneous and cohesive binder structure.

**Figure 4:**
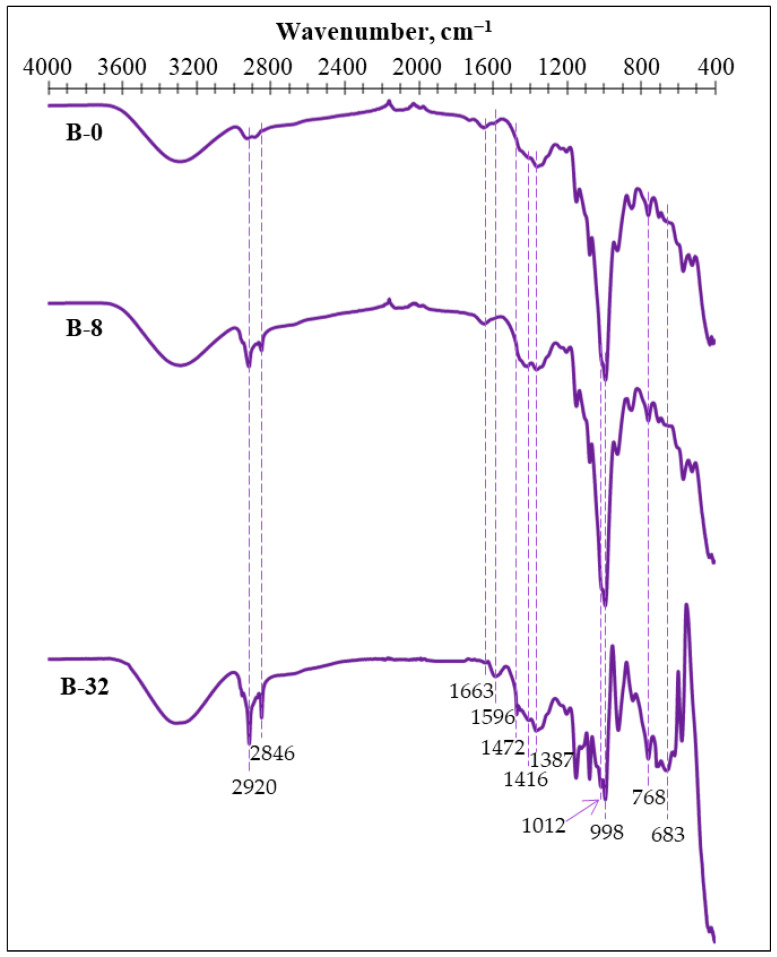
FTIR of obtained potato starch binders.

To characterize the structural changes in the potato starch binder due to the modification of the binder composition, FTIR spectral curves (Figure 4) have been taken for binders, given in Table 1.

In the case of samples B-0 and B-8, the bending of molecular-coordinated water within the SiO_2_^-^ structure appears as a relatively small peak at 1663 cm^−1^ [32]. While in the case of B-32, this band moves to 1596 cm^−1^ due to composition changes (Table 1). The bands at 1012–998 cm^−1^ can be identified as the stretching vibration of CH_2_–O–CH_2_, but the small band at 819 cm^−1^ represents the CH_2_–O–CH_2_ ring mode stretching vibration [33].

The double sharp peaks appearing in B-8 and B-32 at 2920 and 2846 cm^−1^, respectively, is considerable compared to the aliphatic C-H stretching vibration of the paraffin in the PCM additive, but bands detected at 683 cm^−1^ and 768 cm^−1^ can be associated with the C-H out-of-plane wagging vibration of a single-substitute ring [34].

**Figure 5:**
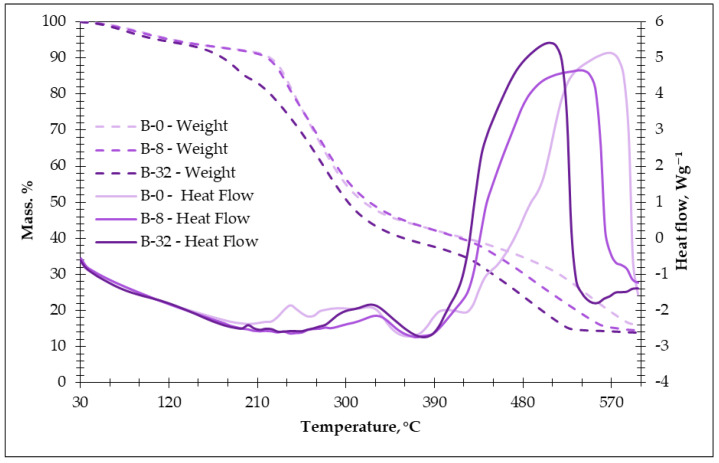
TGA/DSC of obtained potato starch binder.

The thermogravimetric analysis and differential scanning calorimetry (TGA/DSC) was used to study the thermal transformation occurring in the process of heating (Figure 5).

Water evaporation from the samples is observed during stage I of the process at a temperature of 120 °C with a mass loss of -3.8% for the BC-0 sample and 4.2% and 4.6% for the BC-8 and BC-32 samples. The amount of water introduced with sodium metasilicate affected the weight loss. The same vaporization of glycerol starts at 120 °C. Further, the TGA and DTG curves show two regions of weight loss, which were reflected by two peaks on the DTG curves, pointing out that the starch silication products revealed at least two-step decomposition. There was a characteristic peak of starch in the range of 190–350 °C—the first step of decomposition—and another peak related to a deep thermolysis of starch in the region of 390–580 °C—the second step of decomposition.

The typical DSC curves of potato starch have a decomposition onset temperature of about 290–310 °C [35,36,37]. Another study pointed out that the decomposition of starch took place at 277 °C with the loss of 26.73% of its weight within the range of 161–352 °C. It is known that SS degrades at a temperature interval of (150–300 °C) [38]. On heating up to 174 °C, sodium metasilicate gradually lost two water molecules [39]. The sodium silicate, initially being amorphous, starts polymerization and generates crystals of β-Na_2_Si_2_O_5_ at about 400 °C and crystallizes the SiO_2_ modification cristobalite at about 600 °C, which coexists along with β-Na_2_Si_2_O_5_ up to 700 °C [40].

Additionally, during the first step of decomposition in the temperature region of 194–246 °C, glycerol readily decomposes [41].

A large exotherm at approximately 200–320 °C (for BC-0 sample) represents the decomposition of starch in the presence of sodium metasilicate solution. For the BC-8 and BC-32 samples, this effect appeared in the range of 250–330 °C and 260–390 °C, and this can be influenced by the higher amount of the PCM in the composition and polymerization of sodium metasilicate solution. Phase evolution on the heat treatment of sodium silicate water glass was studied [40]. The mass loss during this exotherm period was 58% for BC-0 and 60% and 64% for BC-8 and BC-32 compositions. The decomposition peak is placed at a higher temperature with an increase in the PCM amount in the composition. Most of the organic PCM materials are not stable at higher temperatures because of the covalent bonds. This can happen due to the decomposition of PCM polyurethane capsules [42], which takes place at 200–325 °C, and further burning out of paraffin wax.

The second step of the decomposition of silicated starch, representative of degradation with the weight loss of approximately 76–78%, is in the temperature range of 390–580 °C. It can be noticed that as the amount of the PCM increases, the temperature limits of the second exothermic effect decrease from 580 °C to 500 °C. This can be due to sodium silicate hydrate’s faster dehydration due to a higher amount of burned PCM, which speeds up the reaction.

### 3.2. The Behavior of Binder Bio-Composites

**Figure 6:**
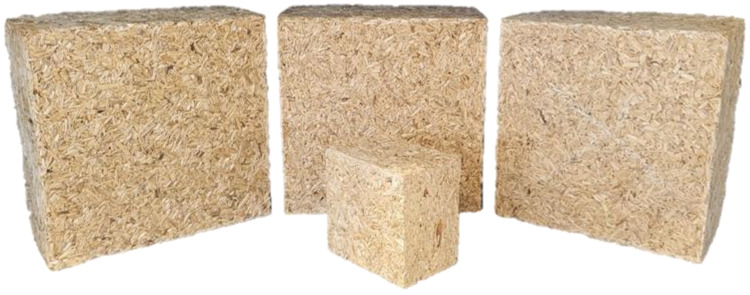
Obtained bio-composites.

In this study, five compositions of bio-composites have been prepared using a potato starch binder with a different amount of the PCM additive (respectively, from 0% to 32% potato starch mass in the composition, Table 2). Figure 6 shows the bio-composites produced. As can be seen in the figure, the material has a uniform structure, with the hemp shive oriented mainly perpendicular to the direction of sample embedment. The resulting materials are relatively light and easy and convenient to handle (e.g., to cut). When the bio-composites are processed (i.e., by cutting), it can be observed that the addition of the PCM significantly improves the structural stability of the material.

**Figure 7:**
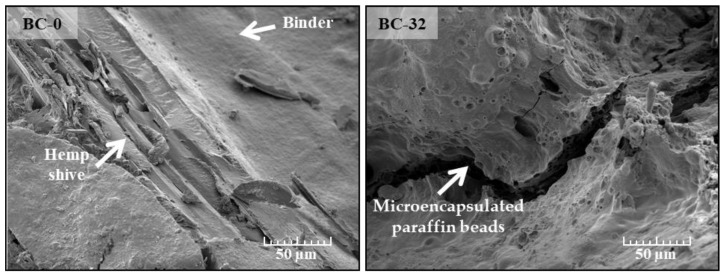
Microstructure of BC-0 and BC-32.

The SEM images (Figure 7) illustrate the microstructural properties of the resulting bio-composites, i.e., hemp shive coated with the modified potato starch binder without (left B-0) and with (right B-32) the PCM additive.

In Figure 7 (left), the microstructure of the B-0 appears more layered, with the binder coating forming relatively thin, layered structures on the surface of the hemp shive. These layers appear to adhere to the hemp shive but remain clearly separated, so the binder may not have fully penetrated the structure of the hemp shive. This type of bonding between the binder and the aggregate particle may result in a weaker adhesion between the binder and the hemp shive, which directly affects the mechanical properties of the resulting bio-composite, such as compressive and bending strength.

However, Figure 7 (right) shows that the morphology of B-32 is significantly different, i.e., the binder has formed a thicker and more homogeneous layer on top of the hemp shive. It can be seen that the surface is more homogeneously covered, with less visible boundaries between the binder and the aggregate. The microstructure is denser, and in some places, the granular structure of the PCM in the binder is visible. It is possible that the PCM helps to fill voids in the bio-composite, thus improving the overall quality of the binder coating and achieving better adhesion between the binder and aggregate. A denser and more homogeneous binder may reduce the porosity and therefore increase the bulk density of the bio-composite, which may have a positive effect on the mechanical properties of the resulting bio-composite.

**Figure 8:**
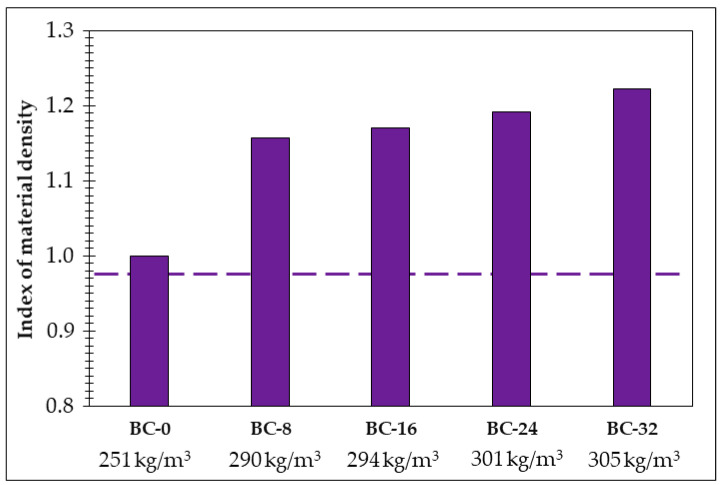
The index of the material density of the obtained bio-composites depending on the PCM amount in the composition.

The bulk density index of the obtained bio-composites, depending on the composition (Table 2), is given in Figure 8. As can be seen in the figure, the PCM additive ensures the increase in the bio-composite bulk density from 16 to 22%. An increase in the amount of the PCM additive in the composition directly correlates with an increase in the bulk density of the resulting bio-composite.

**Figure 9:**
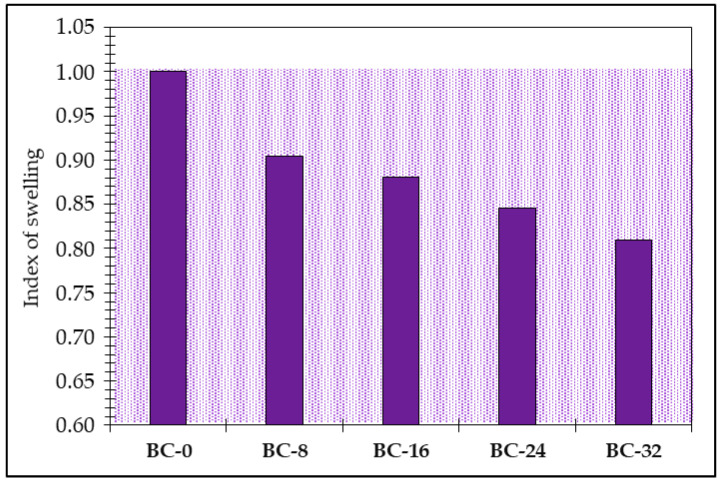
The index of swelling of the obtained bio-composites depending on the PCM amount in the composition (pale purple area—region indicating improved swelling performance compared to the reference sample BC-0).

The effect of the PCM additive on the structure of the obtained bio-composite, as seen in both the SEM images (Figure 7) and bulk density (Figure 8) results, is reflected in the swelling results as well (Figure 9).

Adding the PCM additive to the modified potato starch binder positively affects the obtained bio-composite behavior in contact with water, i.e., the PCM additive decreases the material swelling of thickness. As can see in Figure 9, the PCM additive can improve the volumetric stability of the material under the influence of water by 10 to 19%.

**Figure 10:**
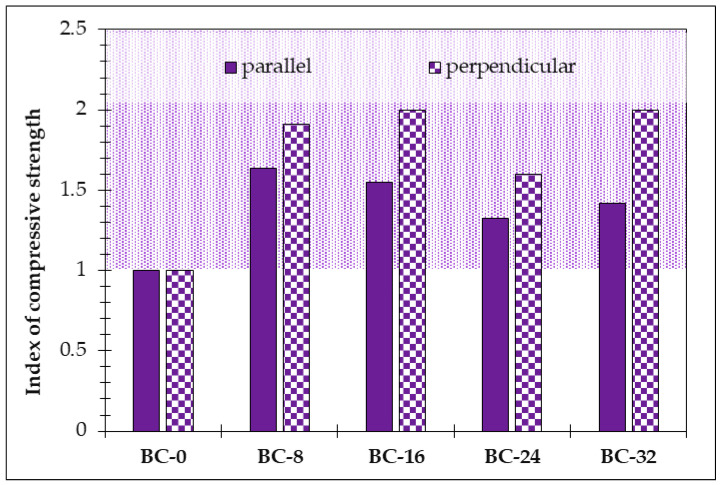
The index of the compressive strength of the bio-composite samples depending on the PCM amount in the composition (pale purple area—region indicating improved compressive strength performance compared to the reference sample BC-0).

The compressive strength results parallel and perpendicular to the direction of production are given in Figure 10. In both directions, the PCM additive improves the compressive strength up to twice (for 33–64% parallel and for 60–100% perpendicular, respectively). From the point of compressive strength, the optimal amount of the PCM additive is 8% potato starch (Table 2), which improves the compressive strength for 90% parallel and for 91% perpendicular.

**Figure 11:**
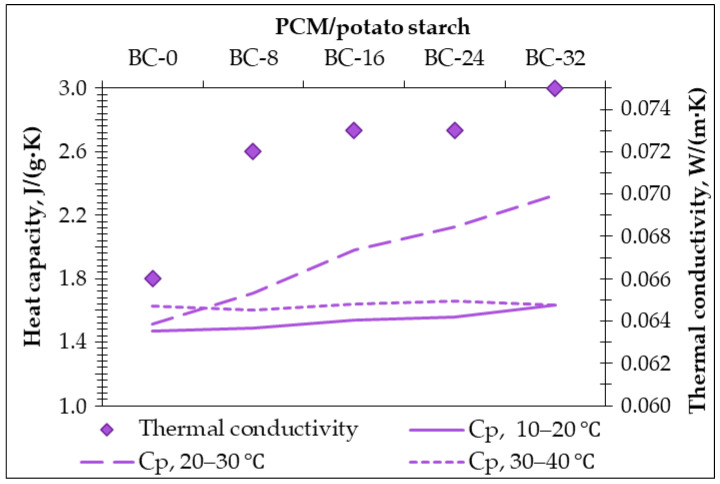
Heat capacity and thermal conductivity of obtained bio-composites.

The obtained bio-composites present the thermal conductivity in the range of 0.066 to 0.075 W/(m·K). As can be seen in Figure 11, the thermal conductivity increases by increasing the PCM additive in the composition (Table 2).

The specific heat capacity (Cp) has been evaluated for the obtained bio-composites in different temperature ranges (i.e., 10–20 °C, 20–30 °C, and 30–40 °C) (Figure 11).

The specific heat capacity of the bio-composite without the PCM additive (B-0) reaches 1.469 J/g·K in the temperature range of 10 to 20 °C; in 20 to 30 °C, it increases for 3%, but in 30 to 40 °C, it increases for 10%. By adding the PCM additive, the behavior of the bio-composites changes; for all compositions, the specific heat capacity is relatively stable in the temperature ranges of 10–20 °C and 30–40 °C. Meanwhile, in the temperature range of 20–30 °C, the specific heat capacity increases linearly up to 54% with increasing amounts of the PCM additive in the composition.

**Figure 12:**
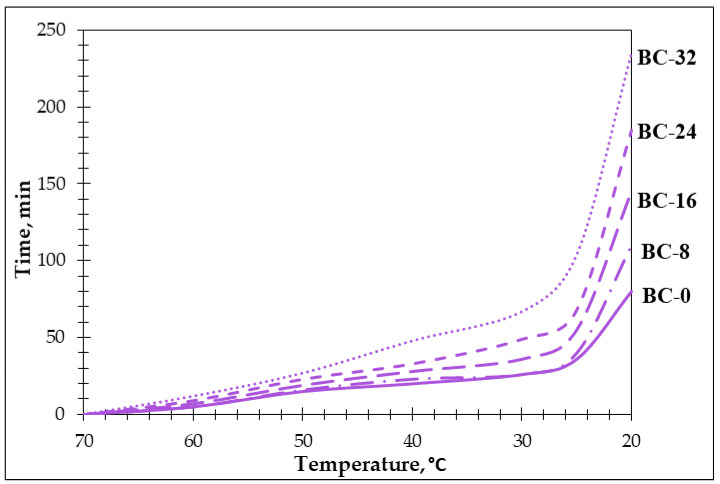
The cool-down time of the obtained bio-composites.

The effectiveness of PCM integration is significantly influenced by the location and outdoor temperature. Research indicates that building surfaces in different climatic zones can reach temperatures even up to 60–70 °C during summer. The influence of a PCM on heat transfer through external wall facades can be evaluated. To examine the impact of a PCM addition, samples were preheated to various temperatures simulating the 70 °C conditions observed on building wall surfaces during hot seasons, and the temperature variations during the cooling phase were monitored. The results of the test are given in Figure 12.

The temperature transition variations inside the reference samples (B-0, respectively) and samples with the PCM additive (B-8, B-16, B-24, and B-32) have been recorded until they reached 20 °C (with a 10 °C step until 30 °C and 5 °C until 20 °C). Though the pre-treatment temperature of the obtained bio-composites was the same, the amount of the PCM in the samples had an impact on the sample’s cooling rate temperature (Figure 12). The results clearly show that PCM content in the sample influences the cooling time. B-0 reached 60 °C in 5 min while BC-32 in 12 min, respectively; the 32% PCM additive ensures about 1.5 times longer a cooling time. With similar behavior during all the tests but in the temperature range below 30 °C, the difference between the reference (B-0) and samples with the PCM additive (B-8, B-16, B-24, and B-32) is more visible.

## 4. Discussion

During this study, it is proven that phase change material (PCM) additives could be used to improve the properties of both modified potato starch binders and hemp shive-based bio-composites bounded by a modified potato starch binder. The influence on microstructure, thermal properties, mechanical performance, and volumetric stability in contact with water highlights the potential of PCM-enhanced materials for use in sustainable construction applications.

The rheological properties of the modified potato starch binder have exhibited notable changes with the inclusion of a PCM. The viscosity of the gelatinized starch decreased by up to 21.6% with higher PCM additive content (i.e., 32% starch). The reduction in viscosity can be attributed to several factors related to the interaction of PCM microcapsules with the starch matrix. The microencapsulated PCM particles act as physical fillers that disrupt the continuity of the starch network, reducing interparticle friction and enhancing matrix flowability. Additionally, the hydrophobic nature of the paraffin-based PCM reduces the interaction between water molecules and starch polymers during gelatinization, thereby limiting the extent of network formation and reducing overall viscosity. Similar effects have been observed in systems where inorganic salts or hydrophobic materials interfere with starch hydration and swelling [30,31]. The encapsulated PCM may also contribute to lubrication within the slurry, further facilitating flow and reducing energy dissipation during mixing. These interactions collectively ensure improved workability and better slurry uniformity, which are critical for effective mixing and processing.

The thermal analysis (TGA/DSC) has confirmed the enhanced stability ensured by the PCM additive to the modified potato starch binder. The onset of decomposition shifts to higher temperatures with increased PCM content, suggesting improved thermal resistance. Additionally, the specific heat capacity of the obtained bio-composites increases linearly within the PCM phase-change temperature range (20–30 °C), demonstrating the effectiveness of the PCM in enhancing the thermal storage capacity of the bio-composites. The prolonged cooling time observed of bio-composites with the PCM additive—up to three times longer compared to reference samples—further validates their potential for energy-efficient thermal regulation in building envelopes.

The mechanical performance (i.e., compressive strength) of bio-composites improved significantly by adding the PCM additive to the composition, with optimal performance achieved at 8% PCM additive. This enhancement aligns with the observed denser microstructure and improved binder–aggregate interaction of bio-composites with a PCM additive.

The observed increase in specific heat capacity, up to 54% in the 20–30 °C range (Figure 11), highlights the significant potential of PCM-enhanced bio-composites for thermal energy storage. To better contextualize this improvement, conventional building materials such as concrete, gypsum, and brick typically exhibit specific heat capacities in the range of 0.84 to 1.00 J/g·K [19,20]. In contrast, the specific heat capacity of bio-composites with PCM in this study reaches 2.26 J/g·K in the 20–30 °C range, demonstrating a more efficient heat storage capability. This enhancement enables the material to absorb and release thermal energy more effectively, reducing temperature fluctuations and improving indoor thermal comfort. By comparison, common thermal insulators like polystyrene and mineral wool have specific heat capacities around 1.3 J/g·K but lack the phase change properties that further enhance thermal regulation. Thus, the significant increase observed with PCM bio-composites highlights their dual benefit of latent heat storage and improved overall heat capacity, making them superior candidates for sustainable building applications.

Despite the promising results, challenges such as the higher cost of a PCM and its integration into large-scale construction applications must be addressed. Future studies could explore cost-effective PCM alternatives and optimize the proportion of the PCM for various climatic conditions and building requirements.

## 5. Conclusions

This study confirms that integrating phase change materials (PCMs) into modified potato starch binders and hemp shive bio-composites enhances their performance, offering a sustainable alternative to conventional building materials.

The addition of the PCM significantly reduced the viscosity of the gelatinized starch by up to 21.6%, improving the binder’s workability and flow properties during the mixing process. Enhanced flowability ensures better aggregate dispersion and more uniform bio-composite formation, contributing to improved processing efficiency.

The inclusion of the PCM resulted in a denser, more cohesive binder matrix, leading to stronger adhesion between the binder and hemp shive aggregate. As a result, the compressive strength of the bio-composites increased significantly, with optimal performance achieved at 8% PCM content, doubling the compressive strength compared to the control sample.

The PCM additive improved thermal storage capacity, with a 54% increase in specific heat capacity within the 20–30 °C range. This enhancement enables efficient heat absorption and controlled release, leading to prolonged cooling times, up to three times longer than those of the reference samples. These properties demonstrate the bio-composites’ potential for passive thermal regulation and energy savings in buildings.

Furthermore, the PCM-modified bio-composites exhibited improved volumetric stability, with reduced water-induced swelling, ensuring better long-term performance in humid environments. Future research could focus on optimizing PCM content based on different climatic conditions and exploring cost-effective alternatives for large-scale applications.

## Figures and Tables

**Table 1:** Compositions of potato starch binders, mass parts.

Composition	Potato Starch	Sodium Metasilicate Solution	Glycerol	PCM	Water
**B-0**	1.0	0.6	0.3	0.00	4.7
**B-8**	1.0	0.6	0.3	0.08	4.7
**B-32**	1.0	0.6	0.3	0.32	4.7

**Table 2:** Compositions of bio-composites, mass parts.

Composition	Raw Materials for Binder, Mass Parts				PCM	Hemp Shives
	Potato Starch	Sodium Metasilicate Solution	Glycerol	Water		
**BC-0**	1.0	0.6	0.3	4.7	0.00	3.3
**BC-8**	1.0	0.6	0.3	4.7	0.08	3.3
**BC-16**	1.0	0.6	0.3	4.7	0.16	3.3
**BC-24**	1.0	0.6	0.3	4.7	0.24	3.3
**BC-32**	1.0	0.6	0.3	4.7	0.32	3.3

## Data Availability

The original contributions presented in the study are included in the article; further inquiries can be directed to the corresponding author.
